# Diverse Imaging Methods May Influence Long-Term Oncologic Outcomes in Oligorecurrent Prostate Cancer Patients Treated with Metastasis-Directed Therapy (the PRECISE-MDT Study)

**DOI:** 10.2967/jnumed.124.267586

**Published:** 2024-08

**Authors:** Matteo Bauckneht, Francesco Lanfranchi, Domenico Albano, Luca Triggiani, Flavia Linguanti, Luca Urso, Rosario Mazzola, Alessio Rizzo, Elisa D’Angelo, Francesco Dondi, Eneida Mataj, Gloria Pedersoli, Elisabetta Maria Abenavoli, Luca Vaggelli, Beatrice Detti, Naima Ortolan, Antonio Malorgio, Alessia Guarneri, Federico Garrou, Matilde Fiorini, Serena Grimaldi, Pietro Ghedini, Giuseppe Carlo Iorio, Antonella Iudicello, Guido Rovera, Giuseppe Fornarini, Diego Bongiovanni, Michela Marcenaro, Filippo Maria Pazienza, Giorgia Timon, Matteo Salgarello, Manuela Racca, Mirco Bartolomei, Stefano Panareo, Umberto Ricardi, Francesco Bertagna, Filippo Alongi, Salvina Barra, Silvia Morbelli, Gianmario Sambuceti, Liliana Belgioia

**Affiliations:** 1 Nuclear Medicine, IRCCS Ospedale Policlinico San Martino, Genova, Italy;; 2 Department of Health Sciences, University of Genova, Genova, Italy;; 3 Nuclear Medicine, ASST Spedali Civili di Brescia, Brescia, Italy;; 4 University of Brescia, Brescia, Italy;; 5 Radiation Oncology, ASST Spedali Civili di Brescia, Brescia, Italy;; 6 Nuclear Medicine, Careggi University Hospital, Florence, Italy;; 7 Nuclear Medicine, Ospedale San Donato, Arezzo, Italy;; 8 Nuclear Medicine, Oncological Medical and Specialist Department, University Hospital of Ferrara, Ferrara, Italy;; 9 Advanced Radiation Oncology, IRCCS Sacro Cuore Don Calabria Hospital, Cancer Care Center, Negrar, Italy;; 10 Nuclear Medicine, Candiolo Cancer Institute, FPO–IRCCS, Turin, Italy;; 11 Radiation Oncology, University Hospital of Modena, Modena, Italy;; 12 Radiation Oncology, Careggi University Hospital, Florence, Italy;; 13 Radiotherapy, University Hospital of Ferrara, Ferrara, Italy;; 14 Radiation Oncology, Candiolo Cancer Institute, FPO–IRCCS, Turin, Italy;; 15 Nuclear Medicine, AOU Città della Salute e della Scienza di Torino, University of Turin, Turin, Italy;; 16 Nuclear Medicine, Oncology, and Haematology Department, University Hospital of Modena, Modena, Italy;; 17 Radiation Oncology, Department of Oncology, University of Turin, Turin, Italy;; 18 Medical Oncology 1, IRCCS Ospedale Policlinico San Martino, Genova, Italy;; 19 Radiotherapy, IRCCS Ospedale Policlinico San Martino, Genova, Italy; and; 20 Nuclear Medicine, IRCCS Ospedale Sacro Cuore Don Calabria, Negrar, Italy

**Keywords:** oligorecurrent prostate cancer, metastasis-directed therapy, choline, prostate-specific membrane antigen, PET

## Abstract

Metastasis-directed therapy (MDT) has been tested in clinical trials as a treatment option for oligorecurrent prostate cancer (PCa). However, there is an ongoing debate regarding the impact of using different imaging techniques interchangeably for defining lesions and guiding MDT within clinical trials. **Methods:** We retrospectively identified oligorecurrent PCa patients who had 5 or fewer nodal, bone, or visceral metastases detected by choline or prostate-specific membrane antigen (PSMA) PET/CT and who underwent MDT stereotactic body radiotherapy with or without systemic therapy in 8 tertiary-level cancer centers. Imaging-guided MDT was assessed as progression-free survival (PFS), time to systemic treatment change due to polymetastatic conversion (PFS2), and overall survival predictor. Propensity score matching was performed to account for clinical differences between groups. **Results:** Of 402 patients, 232 (57.7%) and 170 (42.3%) underwent MDT guided by [^18^F]fluorocholine and PSMA PET/CT, respectively. After propensity score matching, patients treated with PSMA PET/CT–guided MDT demonstrated longer PFS (hazard ratio [HR], 0.49 [95% CI, 0.36–0.67]; *P* < 0.0001), PFS2 (HR, 0.42 [95% CI, 0.28–0.63]; *P* < 0.0001), and overall survival (HR, 0.39 [95% CI, 0.15–0.99]; *P* < 0.05) than those treated with choline PET/CT–guided MDT. Additionally, we matched patients who underwent [^68^Ga]Ga-PSMA-11 versus [^18^F]F-PSMA-1007 PET/CT, observing longer PFS and PFS2 in the former subgroup (PFS: HR, 0.51 [95% CI, 0.26–1.00]; *P* < 0.05; PFS2: HR, 0.24 [95% CI, 0.09–0.60]; *P* < 0.05). **Conclusion:** Diverse imaging methods may influence outcomes in oligorecurrent PCa patients undergoing MDT. However, prospective, head-to-head studies, ideally incorporating a randomized design, are necessary to provide definitive evidence and facilitate the practical application of these findings.

## Visual Abstract

**Figure:**
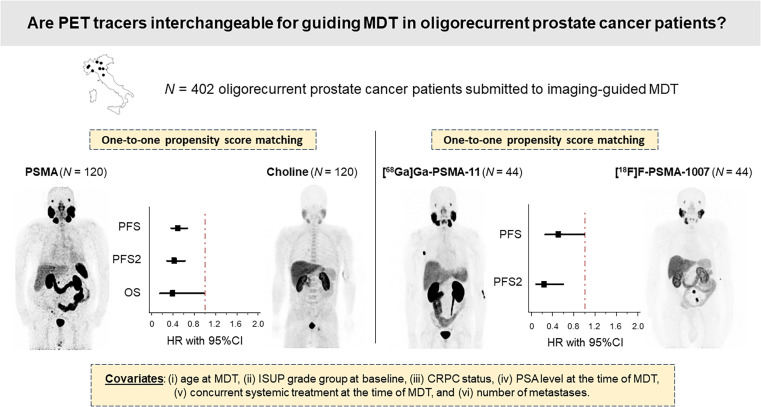

Primary treatment for advanced prostate cancer (PCa) involves androgen deprivation therapy (ADT) (1). However, the effectiveness of ADT is limited and often accompanied by significant side effects (2*,*^3^). Consequently, when metastases are limited in number and location, metastasis-directed therapies (MDTs) using stereotactic body radiotherapy become valuable options, potentially delaying ADT initiation and treatment-related adverse events.

MDT has demonstrated potential in this space in 2 phase II trials (4–6). However, the consistency of these data is debated (7) because imaging technologies were used interchangeably for defining oligometastatic lesions and guiding MDT (4*,*^5^). In this framework, although for many years [^18^F]fluorocholine and [^11^C]C-choline PET/CT have been recommended for PCa restaging, prostate-specific membrane antigen (PSMA)–targeted tracers have recently emerged as more sensitive (1). It is reasonable to expect that more precise disease identification through advanced imaging could increase the proportion of patients receiving comprehensive MDT, potentially leading to improved oncologic outcomes. However, prospective randomized clinical trials evaluating the benefits of treating oligometastases identified by different imaging approaches are still lacking.

Simultaneously, there is increasing debate about which PSMA radiotracer should be preferred. Although [^68^Ga]Ga-PSMA-11 is among the most extensively studied PSMA-targeted ligands, the emergence of several other PSMA ligands, including [^18^F]F-PSMA-1007, has diversified the options available. Recent reports, however, suggest that the higher incidence of unspecific bone uptake associated with [^18^F]F-PSMA-1007 might result in false-positive findings, potentially compromising its accuracy (8*,*^9^).

Considering these aspects, our study was designed to assess the impact of different imaging modalities on guidance of MDT and their effects on oncologic outcomes within a multiinstitutional, real-world cohort of patients with oligorecurrent PCa.

## MATERIALS AND METHODS

### Study Population and Data Collection

We retrospectively analyzed oligorecurrent hormone-sensitive PCa or castration-resistant PCa (CRPC) patients who underwent imaging-guided MDT across 8 Italian tertiary-level cancer centers between July 2012 and May 2023. The inclusion criteria were a histologically confirmed diagnosis of PCa, detection of pelvic or extraregional nodal relapse (M1a) or of bone or visceral metastases (M1b or M1c, respectively) by either choline or PSMA PET/CT, identification of up to 5 metastases by imaging before MDT, treatment with stereotactic body radiotherapy (with or without systemic therapy), and a minimum of 6 mo of clinical follow-up after MDT. The study adhered to the guidelines of the Declaration of Helsinki and was approved by the local ethical committee (registration number 5/2023–DB id 12914). All subjects gave written informed consent.

### Imaging-Guided MDT and Follow-up

PET/CT scans were performed following current guidelines (1*,*^10^). Because of the study’s retrospective design, PET/CT studies were acquired on different scanners, as detailed in Supplemental Table 1 (supplemental materials are available at http://jnm.snmjournals.org). Patients were managed according to current international guidelines (11). After MDT, patients underwent short-term clinical follow-up according to each institutional protocol, with clinical evaluation and a prostate-specific antigen (PSA) blood test every 3–6 mo. Restaging with PET/CT was performed in cases of biochemical progression after MDT. Further MDT was proposed if patients showed oligoprogression after MDT (with <5 new lesions detected outside the irradiated field). Systemic treatments were administered in cases of polymetastatic disease progression, defined as the appearance of more than 5 metastases. Patients with disease progression were followed up for survival status as part of the long-term follow-up.

### Statistical Analysis

Continuous data are expressed as the mean ± SD. Categoric variables were compared using the χ^2^ test, and continuous variables were analyzed using a 1-way ANOVA. When appropriate, post hoc analyses were performed with the Bonferroni method to adjust for multiple comparisons. Statistical significance was set at a *P* value of less than 0.05. To compare treatment groups, we calculated a propensity score using multivariable logistic models, including the type of PET tracer used as the independent variable and factors widely reported to influence outcomes as dependent variables. These variables included age at MDT, International Society of Urological Pathology grade group at baseline, CRPC status, PSA level at the time of MDT, concurrent systemic treatment at the time of MDT, and number of metastases. The resulting propensity score aimed to balance these covariates across treatment groups, thereby reducing selection bias and enabling a more accurate comparison of outcomes. Propensity matching was then applied to create comparable cohorts on a one-to-one basis based on nearest-neighbor matching with a calibration of 0.01. This procedure matches participants from one group to participants from the other group according to the absolute difference between their propensity scores, which must result in the smallest difference to establish a match. Propensity score matching was performed between patients who underwent choline versus PSMA PET/CT–guided MDT and between patients who underwent [^68^Ga]Ga-PSMA-11 versus [^18^F]F-PSMA-1007 PET/CT. Progression-free survival (PFS) was defined as a composite endpoint, as described previously (5*,*^6^). Briefly, it included any of the following criteria: a rise in PSA level of at least 2 ng/dL and 25% above the nadir; radiologic progression; clinical progression; initiation of ADT for any reason; or mortality (5*,*^6^). PFS2 was defined as the interval between imaging time and the systemic treatment change due to polymetastatic conversion. Overall survival (OS) was measured from the initial imaging time to the date of death from any cause. PFS, PFS2, and OS are expressed in months. The Kaplan–Meier method, using the log-rank test, was used to explore differences in PFS, PFS2, and OS among the matched cohorts. A sensitivity analysis using the inverse probability of treatment weighting (12) was applied to confirm the results. A dedicated temporal analysis, conducted via univariate Cox regression, was undertaken to assess the impact of the year of MDT on the study’s endpoints, ensuring our findings’ temporal integrity. Statistical analysis was conducted using SPSS software version 26 (IBM) and MedCalc version 19.4 (MedCalc Software).

## RESULTS

### Patients’ Clinical Characteristics and Imaging Findings

**FIGURE 1.**
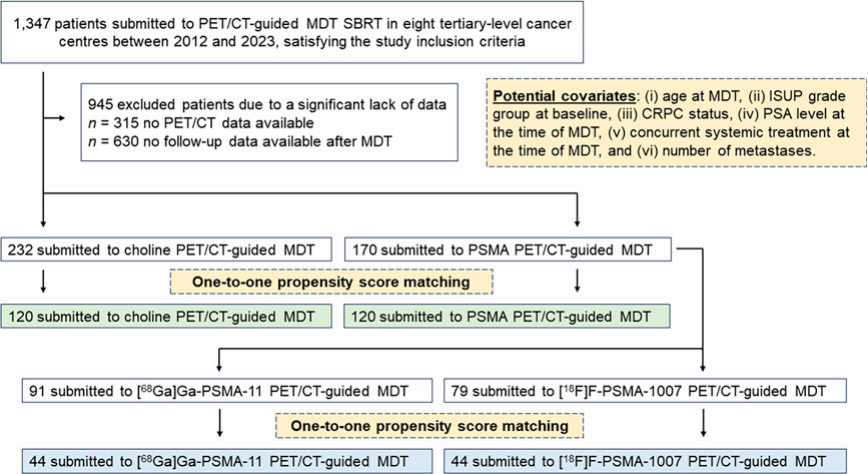
Study design and flowchart of patient selection and matching. ISUP = International Society of Urological Pathology; SBRT = stereotactic body radiotherapy.

We retrospectively selected 402 patients, as detailed in Figure 1. Their clinical characteristics, imaging findings, and MDT parameters are summarized in Table 1. CRPC status was available for 75 patients (18.6%). In most cases (97.8%), patients had 3 or fewer metastases at the pre-MDT imaging. Nodes and bones represented the most frequent metastatic sites. Of 402 patients, 232 (57.7%) and 170 (42.3%) underwent MDT guided by choline and PSMA PET/CT, respectively. All patients who underwent choline PET/CT (*n* = 232) were scanned with [^18^F]fluorocholine, whereas patients who underwent PSMA PET/CT (*n* = 170) were scanned with either [^68^Ga]Ga-PSMA-11 (*n* = 91, 53.5%) or [^18^F]F-PSMA-1007 (*n* = 79, 46.5%).

**TABLE 1.** Clinical, Imaging, and Treatment Characteristics of Patients

Parameter	Data
Preimaging clinical characteristics	
Age (y)	72.60 ± 6.81
Initial AJCC stage	
I	16 (3.98%)
II	91 (22.64%)
III	241 (59.95%)
IV	54 (13.43%)
ISUP grade	
1	62 (15.53%)
2	87 (21.58%)
3	102 (25.26%)
4	59 (14.74%)
5	92 (22.89%)
Primary treatment	
Surgery	322 (80.05%)
Radiotherapy (±ADT)	70 (17.46%)
Medical therapy	10 (2.49%)
CRPC at time of MDT	75 (18.66%)
PSA at time of MDT (ng/mL)	3.21 ± 4.47
Imaging findings	
Imaging-guided MDT	
[^18^F]fluorocholine PET/CT	232 (57.71%)
PSMA PET/CT	170 (42.29%)
Number of metastatic lesions	
1	278 (69.15%)
2	88 (21.89%)
3	27 (6.72%)
4	6 (1.49%)
5	3 (0.75%)
Site of metastases	
Lymph node	283 (70.40%)
Bone	117 (29.10%)
Visceral	2 (0.50%)
MDT parameters and clinical follow-up	
MDT total dose (per lesion)	33.28 ± 4.85
MDT BED (per lesion)	117.67 ± 26.03
Concurrent systemic treatment in addition to MDT	167 (41.54%)
PSA nadir after MDT (ng/mL)	2.07 ± 7.32

AJCC = American Joint Committee on Cancer; ISUP = International Society of Urological Pathology; BED = biologically effective dose.

Qualitative data are number and percentage; continuous data are mean ± SD (*n* = 402).

### Clinical Outcome After MDT According to Imaging Modality

**FIGURE 2.**
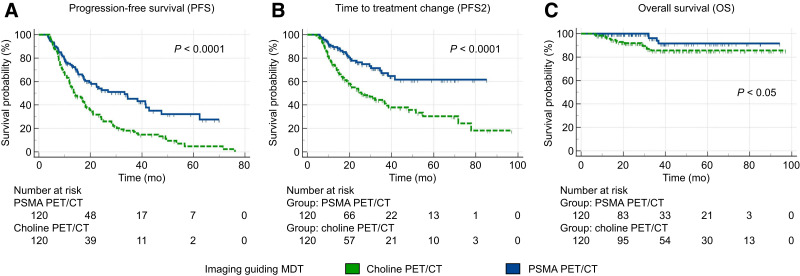
Survival curves according to imaging modality guiding MDT in PSMA and choline PET/CT matched cohorts (*n* = 120).

After propensity score matching, a cohort of 120 patients who underwent choline PET/CT–guided MDT was compared with an equal cohort of 120 patients who underwent PSMA PET/CT–guided MDT. A well-calibrated and discriminative balance between these matched cohorts was documented by the lack of significant differences in any variables across the 2 groups (Table 2). After MDT, patients were clinically and biochemically followed up for a median of 31 mo (95% CI, 28.3–36.2 mo). The median PFS was 17.9 mo (95% CI, 15.2–76.1 mo). Of the 163 (67.9%) patients who experienced progression after MDT, progressors were significantly fewer in the PSMA PET/CT subgroup (59/120, 49.2%) than in the choline subgroup (104/120, 86.7%; *P* < 0.001). Coherently, we observed a significantly longer median PFS in patients undergoing PSMA PET/CT–guided MDT than in those undergoing choline PET/CT–guided MDT (33.2 mo [95% CI, 19.6–41.5 mo] vs. 13.8 mo [95% CI, 11.8–76.1 mo]; hazard ratio [HR], 0.49 [95% CI, 0.36–0.67]; *P* < 0.0001; Fig. 2A). The median PFS2 was 41.5 mo (95% CI, 32.2–77.7 mo). The use of PSMA PET/CT as the guide for MDT was associated with a significantly increased median time to treatment change compared with choline PET/CT (median PFS2 not reached vs. 25.6 mo [95% CI, 19.3–37.7 mo]; HR, 0.42 [95% CI, 0.28–0.63]; *P* < 0.0001; Fig. 2B). The median OS was not reached for the overall cohort or the 2 subgroups. During the follow-up interval, 18 events were recorded, involving 4 and 14 patients in the PSMA and choline PET/CT subgroups, respectively (HR, 0.39 [95% CI, 0.15–0.99]; *P* = 0.014). Figure 2C displays the resulting Kaplan–Meier curves (*P* < 0.05). Notably, patients who underwent different imaging approaches before MDT experienced divergent OS even when OS was measured since PCa diagnosis (HR, 0.31; *P* < 0.05; Supplemental Fig. 1). The sensitivity analyses confirmed these findings (Supplemental Figs. 2–4). The temporal analysis (Supplemental Table 2) revealed that the year of MDT was not a significant predictor of PFS, PFS2, or OS, affirming the temporal robustness of our findings across imaging modalities.

**TABLE 2.** Clinical, Imaging, and Treatment Characteristics of Patients After Propensity Score Matching

Parameter	Overall (*n* = 240)	[^18^F]fluorocholine-guided MDT (*n* = 120)	PSMA-guided MDT (*n* = 120)	*P*
Preimaging clinical characteristics				
Age (y)	72.07 ± 6.55	71.71 ± 6.87	72.43 ± 6.23	0.397
Initial AJCC stage				
I	10 (4.17%)	5 (4.17%)	5 (4.17%)	1.000
II	59 (24.58%)	26 (21.67%)	33 (27.50%)	0.295
III	142 (59.17%)	72 (60.00%)	70 (58.33%)	0.792
IV	29 (12.08%)	17 (14.17%)	12 (10.00%)	0.322
ISUP grade				
1	31 (12.92%)	17 (14.17%)	14 (11.67%)	0.565
2	65 (27.08%)	32 (26.67%)	33 (27.50%)	0.885
3	55 (22.92%)	25 (20.83%)	30 (25.00%)	0.443
4	39 (16.25%)	18 (15.00%)	21 (17.5%)	0.600
5	50 (20.83%)	28 (23.33%)	22 (18.33%)	0.341
Primary treatment				
Surgery	200 (83.33%)	95 (79.00%)	105 (87.50%)	0.079
Radiotherapy (±ADT)	36 (15.01%)	22 (18.50%)	14 (11.67%)	0.140
Medical therapy	4 (1.66%)	3 (2.50%)	1 (0.83%)	0.313
CRPC at time of MDT	40 (16.67%)	17 (14.17%)	23 (19.17%)	0.254
PSA at time of MDT (ng/mL)	2.66 ± 3.56	2.93 ± 2.44	2.39 ± 1.99	0.243
Imaging findings				
Number of metastatic lesions				
1	183 (76.25%)	91 (75.83%)	92 (76.67%)	0.879
2	38 (15.83%)	17 (14.17%)	21 (17.50%)	0.481
3–5	19 (7.92%)	12 (10.00%)	7 (5.83%)	0.232
Site of metastases				
Lymph node	169 (70.42%)	90 (75.00%)	79 (65.83%)	0.120
Bone	70 (29.17%)	30 (25.00%)	40 (33.33%)	0.157
Visceral	1 (0.42%)	0 (0.00%)	1 (0.84%)	0.315
MDT parameters and clinical follow-up				
MDT total dose (per lesion)	33.42 ± 4.68	33.35 ± 4.17	33.49 ± 5.17	0.824
MDT BED (per lesion)	119.90 ± 26.48	124.60 ± 33.53	116.62 ± 19.97	0.207
Concurrent systemic treatment in addition to MDT	100 (40.83%)	43 (35.80%)	57 (47.50%)	0.067
PSA nadir after MDT (ng/mL)	1.95 ± 7.93	2.67 ± 11.28	1.32 ± 2.48	0.218
Propensity score matching	0.54 ± 0.13	0.54 ± 0.13	0.54 ± 0.13	0.987

AJCC = American Joint Committee on Cancer; ISUP = International Society of Urological Pathology; BED = biologically effective dose.

Qualitative data are number and percentage; continuous data are mean ± SD.

### Outcome in Patients Who Underwent PSMA PET/CT–Guided MDT

**FIGURE 3.**
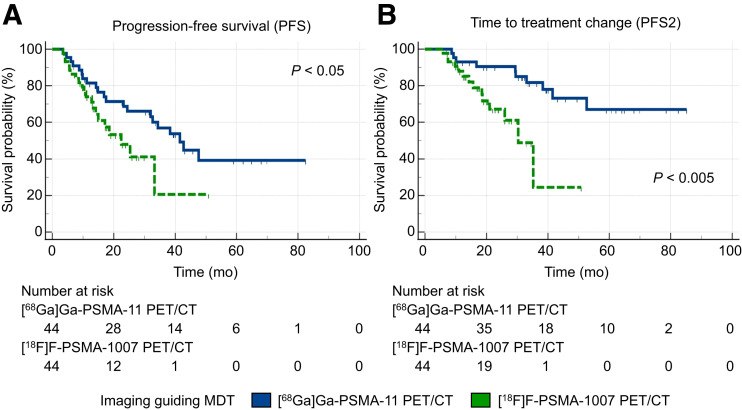
Survival curves according to imaging modality guiding MDT in [^68^Ga]Ga-PSMA-11 and [^18^F]F-PSMA-1007 PET/CT matched cohorts (*n* = 44).

We subsequently compared 2 propensity score–matched cohorts of patients who underwent MDT guided by either [^68^Ga]Ga-PSMA-11 or [^18^F]F-PSMA-1007 PET/CT. The 2 cohorts, consisting of 44 patients, demonstrated well-balanced clinical, imaging, and treatment characteristics (Table 3). The nadir PSA serum level after MDT was significantly lower in patients who underwent [^68^Ga]Ga-PSMA-11 than in those who underwent [^18^F]F-PSMA-1007 (0.53 ± 0.91 vs. 1.69 ± 2.23 ng/mL; *P* < 0.005). Moreover, the use of [^68^Ga]Ga-PSMA-11 as the guide for MDT was associated with significantly increased median PFS (41.5 mo [95% CI, 24.4–47.6 mo] vs. 22.4 mo [95% CI, 14.1–33.2 mo]; HR, 0.51 [95% CI, 0.26–1.00]; *P* < 0.05; Fig. 3A) and median PFS2 (not reached vs. 30.3 mo [95% CI, 21.0–35.2 mo]; HR, 0.24 [95% CI, 0.09–0.60]; *P* < 0.005; Fig. 3B) compared with [^18^F]F-PSMA-1007. The sensitivity analyses confirmed these findings (Supplemental Figs. 5–6). Differences in OS were not assessed in this subgroup, as no events were recorded in patients undergoing [^18^F]F-PSMA-1007 PET/CT–guided MDT. The temporal analysis confirmed the temporal robustness of our observations (Supplemental Table 3).

**TABLE 3.** Clinical, Imaging, and Treatment Characteristics of Patients Undergoing [^18^F]F-PSMA-1007 or [^68^Ga]Ga-PSMA-11 PET/CT–Guided MDT After Propensity Score Matching

Parameter	Overall (*n* = 88)	[^18^F]F-PSMA-1007–guided MDT (*n* = 44)	[^68^Ga]Ga-PSMA-11–guided MDT (*n* = 44)	*P*
Preimaging clinical characteristics				
Age (y)	73.07 ± 6.42	73.2 ± 7.23	72.94 ± 5.56	0.850
Initial AJCC stage				
I	4 (4.55%)	3 (6.82%)	1 (2.27%)	0.308
II	22 (25.00%)	9 (20.45%)	13 (29.55%)	0.327
III	54 (61.36%)	26 (59.09%)	28 (63.64%)	0.663
IV	8 (9.09%)	6 (13.64%)	2 (4.54%)	0.140
ISUP grade				
1	10 (11.36%)	4 (9.09%)	6 (13.64%)	0.504
2	19 (21.59%)	10 (22.73%)	9 (20.45%)	0.796
3	25 (28.41%)	12 (27.27%)	13 (29.55%)	0.814
4	19 (21.59%)	10 (22.73%)	9 (20.45%)	0.796
5	15 (17.05%)	8 (18.18%)	7 (15.91%)	0.778
Primary treatment				
Surgery	74 (84.09%)	34 (77.27%)	40 (90.91%)	0.082
Radiotherapy (±ADT)	14 (15.91%)	10 (22.73%)	4 (9.09%)	0.082
CRPC at time of MDT	15 (17.05%)	7 (15.91%)	8 (18.18%)	0.778
PSA at time of MDT (ng/mL)	2.42 ± 5.01	2.27 ± 3.80	2.58 ± 6.05	0.769
Imaging findings				
Number of metastatic lesions				
1	69 (78.41%)	34 (77.27%)	35 (79.55%)	0.796
2	13 (14.77%)	6 (13.54%)	7 (15.91%)	0.755
3–5	6 (6.82%)	4 (9.09%)	2 (4.54%)	0.400
Site of metastases				
Lymph node	59 (67.04%)	27 (61.36%)	32 (72.73%)	0.259
Bone	28 (31.82%)	16 (36.36%)	12 (27.27%)	0.367
Visceral	1 (1.14%)	1 (2.27%)	0 (0.00%)	0.318
MDT parameters and clinical follow-up				
MDT total dose (per lesion)	34.02 ± 4.86	33.84 ± 5.22	34.20 ± 4.51	0.731
MDT BED (per lesion)	117.89 ± 20.18	115.40 ± 11.77	120.11 ± 25.64	0.492
Concurrent systemic treatment in addition to MDT	29 (32.96%)	14 (31.82%)	15 (34.09%)	0.822
PSA nadir after MDT (ng/mL)	1.093 ± 1.77	1.70 ± 2.24	0.53 ± 0.91	0.003
Propensity score matching	0.52 ± 0.15	0.52 ± 0.15	0.52 ± 0.15	0.988

AJCC = American Joint Committee on Cancer; ISUP = International Society of Urological Pathology; BED = biologically effective dose.

Qualitative data are number and percentage; continuous data are mean ± SD.

## DISCUSSION

There is considerable uncertainty in interpreting and applying clinical trial findings for oligorecurrent PCa, mainly attributable to varied imaging approaches. Table 4 summarizes existing real-world retrospective studies (13–17) comparing the efficacy of different imaging-guided MDT approaches. Beyond providing a larger patient sample, our multiinstitutional observational study contributes additional valuable insights.

**TABLE 4.** Overview of Previous Studies Regarding SBRT-Delivered MDT Guided by Different Imaging Techniques in Oligorecurrent PCa

Author	Oligometastatic patients (*n*)	Disease phase*	Imaging-guided MDT	Treatment received	Median follow-up (mo)	Endpoint	Result
Schmidt Hegemann, 2020 (13)	272 (subgroup analysis)	HSPC (NA)	[^68^Ga]-PSMA-11 vs. [^18^F]fluorocholine or [^11^C]C-choline PET/CT	SBRT ± ADT	30	bPFS	Imaging-guided MDT did not predict bPFS
Mazzola, 2021 (14)	88	HSPC (≤3)	[^68^Ga]Ga-PSMA-11 vs. [^18^F]fluorocholine PET/CT	SBRT	25	dPFS, ADT-FS	Imaging-guided MDT predicted ADT-FS but not dPFS
Deijen, 2021 (15)	50	HSPC (≤4)	[^68^Ga]Ga-PSMA-11 vs. [^18^F]F-methylcholine PET/CT	SBRT ± ADT	24.3	bPFS, ADT-FS	Imaging-guided MDT predicted bPFS and ADT-FS
Lanfranchi, 2023 (16)	37	HSPC or CRPC (≤5)	[^68^Ga]Ga-PSMA-11 vs. [^18^F]fluorocholine PET/CT	SBRT ± ADT	40.9	PFS (composite)	Imaging-guided MDT predicted PFS
Metz, 2023 (17)	123	HSPC (≤5)	[^68^Ga]Ga-PSMA-11 vs. [^18^F]fluorocholine PET/CT	SBRT ± ADT	42.2	bPFS, ADT-FS	Imaging-guided MDT predicted bPFS and ADT-FS

* Data in parentheses are number of metastatic lesions.

HSPC = hormone-sensitive prostate cancer; NA = not applicable; SBRT = stereotactic body radiotherapy; bPFS = biochemical PFS; dPFS = distant PFS; ADT-FS = ADT-free survival.

First, whereas previous studies focused on PFS, we observed differences in PFS2 and OS. Subject to confirmation by further studies, this may represent a relevant step forward in MDT validation, as PFS is a questionable surrogate of OS (18). A recent study suggested that the oligometastatic state defined by PSMA PET/CT may represent a less aggressive disease with slower progression, as it is associated with fewer high-risk DNA mutations (19*,*^20^). However, this finding should be interpreted cautiously, considering the evolving landscape of PCa treatment, where advancements in systemic therapies and radiation techniques during the study period may influence outcomes. We used temporal analyses to investigate these effects, yet the potential for residual confounding remains. Future prospective studies are essential to disentangle the specific impact of imaging modalities from these treatment advancements, ensuring a clearer understanding of their comparative effectiveness. Moreover, the higher sensitivity of PSMA PET/CT imaging likely leads to earlier detection of metastatic disease than is possible with choline PET/CT. Identifying metastases earlier introduces a potential lead-time bias known as the Will Rogers phenomenon (21). This occurs when a patient’s disease is reclassified using more sensitive diagnostic tools. With earlier metastasis identification, the interval from imaging to treatment alteration or death may appear prolonged, even though the patient’s life-span remains unchanged. Thus, the observed increase in survival could be attributed to early detection rather than an actual prolongation of life. However, in an exploratory analysis, we observed a difference in OS from the initial diagnosis of PCa rather than from the imaging time. On this basis, we can assume that our findings are not purely the result of the lead-time bias. Ongoing prospective randomized phase III trials (NCT03582774, NCT03762759, and NCT04557501, with estimated completion dates in 2023, 2025, and 2028, respectively) will further address these issues, providing more robust evidence on the topic.

Interestingly, we observed a hierarchy between PSMA-targeted radiopharmaceuticals in differentiating the PSA nadir after therapy and the oncologic outcome of patients who underwent MDT under the guidance of [^68^Ga]Ga-PSMA-11 or [^18^F]F-PSMA-1007. Only a few head-to-head studies comparing these 2 radiotracers are currently available in the literature, mainly from the diagnostic accuracy point of view (22). In a prospective cross-over study on 50 patients, [^18^F]F-PSMA-1007 provided more equivocal results than [^68^Ga]Ga-PSMA-11 (23). Seifert et al. used [^68^Ga]Ga-PSMA-11 as part of a reference standard for [^18^F]F-PSMA-1007 PET/CT bone-uncertain findings (24). An ongoing randomized comparative trial is assessing the noninferiority of [^18^F]F-PSMA-1007 to [^68^Ga]Ga-PSMA-11 (25). To the best of our knowledge, the present study is the first to observe a difference in clinical outcomes in patients managed under the guidance of the 2 tracers. One possible explanation for our findings is the propensity of [^18^F]F-PSMA-1007 to exhibit unspecific bone uptake, potentially leading to false-positive results. Mistaking unspecific uptake for metastatic lesions could result in inappropriate targeting during stereotactic body radiotherapy, thereby affecting the tracer’s effectiveness in guiding MDT. The literature emphasizes the need for sophisticated training in interpreting [^18^F]F-PSMA-1007 PET/CT images (26), pointing to a steeper learning curve and potential variability in physician interpretations. This is especially relevant in nuclear medicine facilities that perform a high volume of [^18^F]F-PSMA-1007 PET/CT scans, for which the understanding and interpretation of bone uptake are in constant evolution. This evolution suggests that future MDT outcomes may vary as methodologies and interpretive approaches adapt to these insights. On the one side, this dynamic underlines a limitation of our study, as the lack of a central imaging review may have introduced heterogeneity in interpretations and potentially affected MDT efficacy. On the other hand, it also underscores a practical challenge in achieving consistent readings across different observers when using this tracer in real-world settings. Further research using a more refined methodology is essential to investigate these concerns thoroughly.

It is important to acknowledge several further limitations of our study. The retrospective and observational design of the study might have resulted in limited statistical power. Additionally, although propensity score matching aimed to reduce heterogeneity in clinically relevant prognostic parameters between patient groups, it may not have completely addressed all disparities. In particular, although not significantly different, we observed a discernible trend toward more frequent use of concurrent ADT in addition to MDT between the PSMA and choline PET/CT matched cohorts. Moreover, we did not consider the type and duration of ADT before MDT in the matching process. Altogether, these limitations prevent drawing a secure causative relationship between the observed differences in oncologic outcome and imaging methods. Therefore, further studies with appropriate methodologies are needed in this field. Nevertheless, the retrospective design was essential for conducting a real-world study, mirroring actual clinical practices and patient care, and providing the advantages of a less selected patient population and more generalizable results. Lastly, in response to the growing interest in integrating systemic therapies with MDT in the CRPC setting (7), we included oligorecurrent CRPC patients in our study. A dedicated subanalysis for CRPC patients could have provided further insights. However, it was not feasible to apply propensity score matching to CRPC patients because of insufficient statistical power. Additional studies are needed to address this point.

## CONCLUSION

Diverse imaging methods may influence outcomes in patients with oligometastatic PCa undergoing MDT. However, prospective head-to-head studies, ideally incorporating a randomized design, are necessary to provide definitive evidence and facilitate the practical application of these findings.

## DISCLOSURE

This work was performed within the framework of the project “RAISE—Robotics and AI for Socioeconomic Empowerment” and has been supported by European Union–NextGenerationEU and by the Italian Ministry of Health (5 × 1000 funds 2020 and Ricerca Corrente Funds 2022 granted to Matteo Bauckneht). Matteo Bauckneht reports personal fees from AAA and GE Healthcare outside the submitted work. No other potential conflict of interest relevant to this article was reported.

## Notes

KEY POINTS **QUESTION:** Are PET tracers interchangeable for guiding MDT in oligorecurrent PCa patients? **PERTINENT FINDINGS:** We observed increased PFS, PFS2, and OS in oligorecurrent PCa patients treated with MDT guided by PSMA PET/CT as opposed to choline PET/CT. A hierarchy among PSMA-targeted radiopharmaceuticals was also observed, favoring [^68^Ga]Ga-PSMA-11 over [^18^F]F-PSMA-1007. **IMPLICATIONS FOR PATIENT CARE:** The choice of PET tracer may influence oncologic outcomes in PCa patients with limited metastases treated with MDT. Prospective, randomized, head-to-head studies are necessary to establish these findings conclusively.
